# Trends in Low-Value Care Following a Payer-Initiated Audit and Feedback Intervention: A Retrospective Analysis in Hospitals With Varying Payment Models

**DOI:** 10.34172/ijhpm.9060

**Published:** 2026-08-18

**Authors:** Vera de Weerdt, Xander Koolman, Hanna C. Willems, Sjoerd Repping, Piet A. Stam, Jacques van Limbeek, Eric J.E. van der Hijden

**Affiliations:** ^1^Department of Health Economics, School of Business and Economics, Vrije Universiteit, Amsterdam, The Netherlands.; ^2^Amsterdam University Medical Center, Amsterdam, The Netherlands.; ^3^Department of Geriatrics, Amsterdam University Medical Center, Amsterdam, The Netherlands.; ^4^Leading the Change, Zorgevaluatie Nederland, The Netherlands.; ^5^National Healthcare Institute, Diemen, The Netherlands.; ^6^ONVZ Health Insurance, Houten, The Netherlands.; ^7^Zilveren Kruis Health Insurance, Zeist, The Netherlands.

**Keywords:** Low-Value Care, Appropriate Care, Quality Improvement, Audit & Feedback, Payment Models, Financial Incentives

## Abstract

**Background:**

Reducing low-value care (LVC) remains a significant challenge in healthcare systems, despite efforts for de-implementation. To facilitate de-implementation payers may use provider Audit & Feedback (A&F). This study aims to examine trends in LVC following a payer-initiated A&F program, and to explore whether this association varies across hospital payment models.

**Methods:**

Multilevel logistic regression was used to examine the trend in LVC in hospitals, its correlation with an A&F intervention, and whether this varied with payment models including fee-for-service (FFS), global budget (GB), cost ceiling (CC) and gradual tariff (GT). To assess this, we used hospital level claims data from a large nationwide Dutch health insurer on 70 hospitals in the period 2017-2022. The primary outcome was the yearly proportion of LVC per hospital. Independent variables were time, market share and payment models. We hypothesized that payer-initiated A&F had a stronger impact in hospitals were the payer had a larger market share, and that the effect would be stronger still for hospitals with financial incentives to reduce production.

**Results:**

LVC showed a small, but significant decline over time (odds ratio [OR]: 0.98; 95% confidence level [CI]: 0.98-0.99). LVC tended to be higher in hospitals where the payer had a higher market share (OR: 1.03; 95% CI: 1.00-1.05). The CC model was associated with an increase in LVC (OR: 1.11; 95% CI: 0.99–1.03). The other payment models were associated with a decrease in LVC. The FFS model exhibited the smallest reduction in LVC (OR: 0.98; 95% CI: 0.98-0.99), and the GT model (OR: 1.01; 95% CI: 0.99-1.03) showed similar patterns in LVC. The GB model was associated with the largest reduction in LVC (OR: 0.94; 95% CI: 0.91-0.97).

**Conclusion:**

By itself A&F did not clearly contribute to the decline of the proportion of LVC; combined with volume-constraining financial incentives, it was associated with larger reductions.

## Introduction

**Key Messages**

**Implications for policy makers**

- Payers may promote adoption of Audit & Feedback (A&F) programs to reduce hospital low-value care (LVC).
- A&F alone was not associated with a reduction in LVC, unless combined with volume-constraining financial incentives.
- Reducing LVC is complex; a multifaceted approach may be warranted.

**Implications for the public**

Low-value care (LVC) can lead to unnecessary treatments, avoidable healthcare costs, and potential harm to patients. This undermines quality and efficiency of healthcare systems. Our research shows that a stand-alone carefully designed Audit & Feedback (A&F) program was not associated with a reduction in LVC. Combined with well-aligned financial incentives, we observed a significant reduction as anticipated. Payment models such as global budgets (GBs) provide healthcare professionals with incentives to reduce LVC in order to optimize access and quality of care. This approach prevents administratively involved and costly restrictive measures such as medical pre-authorization.

Low-value care (LVC) continues to pose a significant challenge within healthcare systems, despite ongoing de-implementation efforts.^1-7^ LVC refers to diagnostic or therapeutic treatments that are either ineffective or not cost-effective for their intended indications.^4,8^ Examples commonly cited include the prescription of antibiotics for acute sinusitis,^9^ prostate cancer screening in men over 70 years of age,^10^ and surgical treatments for low back hernias.^11^ Estimates suggest that approximately 30% of care provided in Western countries qualifies as low-value.^12,13^

 The persistence of LVC not only risks patient harm but also represents a misuse of limited healthcare resources.^5,14^ This issue is particularly pressing given the projected increase in resource scarcity driven by aging populations and advances in medical technology.^15^ Addressing LVC is essential to redirect these resources toward high-value care, ensuring a more efficient and impactful allocation of healthcare expenditures.

 Audit & Feedback (A&F) is a widely utilized quality improvement intervention that can effectively promote the de-implementation of LVC.^16,17^ In A&F treatments, healthcare professionals receive feedback on their clinical behavior to encourage alignment with established norms and/or benchmarks. By providing feedback on the level of LVC in their hospital, the A&F intervention aims to motivate professionals to reduce or eliminate LVC. In 2016, one of the largest Dutch health insurers initiated a policy program employing A&F to reduce hospital provision of LVC treatments. These treatments were identified as LVC based on an insurer-conducted assessment of national and international scientific evidence, clinical guidelines, and governmental evaluations and reports. The health insurer incorporated A&F into the annual procurement cycle, providing hospitals with a detailed report that quantified the annual volume of each LVC treatment performed. These reports were shared and discussed with hospital sales departments to highlight areas for improvement. Hospitals were responsible for disseminating the A&F findings to the relevant clinical departments. Aim of the A&F was to motivate hospitals to de-implement LVC in their hospital. There were no financial agreements regarding LVC specifically.

 Simultaneously, the health insurer employed various payment models to influence providers’ production levels. It is well-established that a fee-for-service (FFS) payment model creates an incentive for production and inherently overtreatment and LVC.^18^ Other payment models, such as a global budget (GB) are presumed to offer less incentive for production and thus less incentive for LVC.^18,19^ Therefore, it is plausible that the effectiveness of A&F in promoting de-implementation of LVC varies across hospitals operating under different payment models.

 Four dominant payment models are used in the Netherlands to contract hospitals: (1) FFS, without an expenditure cap. (2) Gradual Tariff (GT), ie, FFS with different tariffs before and after exceeding an expenditure cap. (3) Cost Ceiling (CC), ie, FFS with an expenditure cap. (4) GB, a set budget regardless of actual production. Each of these payment models is designed to influence providers’ volume of care differently.

 The FFS model pays providers for each episode of care without any cap, thus they experience a financial incentive to produce higher volumes of care.^18^ On the other end, the GB model gives providers a guaranteed budget, regardless of hospital production. Thereby, the GB model incentivizes providers to provide less care, to save costs.^18,19^ In between are the GT and the CC model. The CC model also pays providers for cost-per-episode, but only up to a predetermined expenditure cap.^18,20^ Thus, in a CC model providers have a strong incentive to produce up to the cap, but an incentive to limit production over the cap. The GT model also pays providers for cost-per-episode but applies different prices before and after reaching an expenditure cap, usually prices are reduced for care provided both before and beyond the expenditure cap.^18^ Thus, in the GT model providers also experience a strong incentive to produce up to the cap, but a smaller incentive for production over the cap. This model is applied when there is uncertainty on the expected volume in a year and this model mitigates the volume risk for both provider and payer. The type of payment models for a hospital in the Netherlands is traditionally determined for one year, following contract negotiations between the hospital and health insurers acting in the role of payer.

 While the four payment models offer different financial incentives for providers’ productivity, it is unknown whether and how they affect de-implementation of LVC specifically. The “conditional” payment models (the GT, CC, and GB model) are designed to manage the volume of care, but it is unknown whether providers decrease LVC specifically, or whether general volume of care is reduced (ie, high-value care may also decrease). This study aims to examine trends in LVC following a payer-initiated A&F program, and to explore whether this association varies across hospital payment models, including FFS, GB, CC, and GT.

## Methods

###  Design

 We conducted a multilevel logistic regression analysis on health insurance claims data. Claims data from a nationwide operating health insurer with a representative client base and data on all Dutch hospitals was used.^21^ Data was included for the period 2017-2022.

###  Dutch Context

 Dutch healthcare has a multi-payer system in which competing health insurers act as payers, who negotiate contracts with competing providers. This system was introduced in the Netherlands as of 2006 and is referred to as a “managed competition model.” Health insurers can freely select which providers to contract and can negotiate different tariffs for each provider. The Netherlands has universal coverage with mandatory insurance for a standardized basic benefits package only. Under this package, premiums are regulated, there is annual open enrollment and there is mandatory care provision. There is strong competition between health insurers. Health insurers receive financial risk adjustment for their population under the basic benefits package to correct for large population differences. People can choose to apply for additional coverage for a premium price.

 The Netherlands has four major health insurers with nationwide networks and a combined market share of ~85%.^22^ Additionally, there are a few smaller health insurers, of which most have limited geographical coverage.^20^

 Dutch health insurers all employ the four payment models FFS, GT, CC, and GB. However, which payment model or which mix of payment models is used for one hospital differs between payers. Using payment models to influence provider behavior has become increasingly relevant to address the issues of LVC, fragmentation of care and rising healthcare expenditure.^18^

###  Data Source

 This historical cohort study uses historic healthcare claims data provided by one Dutch health insurer. Data comprised annual care volume per hospital, including total hospital care volume and volume of the 13 LVC treatments. Six out of 13 LVC treatments were excluded from statistical analyses, as these were performed in fewer than 50% of hospitals (Supplementary file 1). Data on the seven treatments performed in over 90% of hospitals were used for statistical analyses (Table 1).

**Table 1:** Description of Low-Value Care Treatments Classified by the Health-Insurer

	**LVC Treatment**	**For Indication (Diagnosis)**
1	Injection and denervation techniques	Non-specific low back pain
2	Adenectomy	Otitis media and pulmonary infection
3	Arthroscopy	Meniscus lesion without lock complaints or patellofemoral pain syndrome
4	Hysterectomy	Heavy menstrual bleeding
5	Leg angioplasty	Peripheral artery occlusive disease without supervised leg training following physiotherapy guidelines
6	Light therapy	Indications other than psoriasis, vitiligo, or uremic itch in dialysis patients
7	Subacromial decompression surgery	Subacromial impingement syndrome

Abbreviation: LVC, low-value care.

 Furthermore, data contained information on payment model per hospital per year, market share of the health insurer per hospital per year and type of hospital (academic, teaching or general hospital). All data was aggregated on hospital level and anonymized.

 Data was obtained for the period 2017-2022. The first year in which the health insurer provided A&F to hospitals, motivating them to de-implement LVC, was 2016.

 Data was obtained for all 79 hospitals in the Netherlands, of which 9 hospitals were excluded from statistical analyses for one of the following reasons: the hospital did not produce any of the 13 LVC treatments or the hospital closed/merged within the study period. Additionally, one hospital merged after 2017, thus this hospital was included for 2018-2022. Statistical analyses were performed for 69 hospitals in 2017 and 70 hospitals for all later years.

###  Hypotheses 

 This study uses the following hypotheses:

1. Following the A&F intervention, LVC shows a decreasing trend over time, which is strongest in hospitals where the health insurer has a higher market share.
2. The decreasing trend in LVC is stronger in hospitals with a payment model that has a smaller incentive for production.

###  Variables 

 The outcome variable was the yearly proportion of LVC per hospital, defined as the number of LVC cases divided by the total hospital care volume ^[1]^. LVC cases and total hospital care volume are measured using claims submitted to health insurers, recorded in a DRG-based system specific to the Netherlands. We examine the trend in LVC over time, measured in years. Time is interacted with the variable Market Share, to account for the fact that the effect of the A&F intervention is expected to be larger in hospitals where the health insurer has a larger market share.^23^

 To examine the influence of payment models on the trend in LVC, an interaction between payment model, time and Market Share was used. The payment models included the four models: FFS, CC, GTs, and GB. Each payment model was represented by a dummy variable, with its value indicating either 0 or the market share of the health insurer, expressed as a proportion (eg, 0.2111). Payment models were used to correct for fixed unobserved differences in LVC correlated with these models.

###  Statistical Analysis 

 A multilevel logistic regression model with a binomial distribution and logit link was used, fitted with the glmer function in the *lme4* package (version 2024.04.1+748). The primary outcome was the yearly proportion of LVC per hospital, defined as the number of LVC cases divided by the total number of treatments. Fixed effects included calendar year (continuous), hospital payment model (reference = FFS), their interaction, and hospital market share (continuous). A random intercept for hospital accounted for clustering of observations within hospitals. Effect estimates are presented as odds ratios (ORs) with 95% confidence intervals (CIs).

 First, we analyzed the trend of LVC over time and whether this trend differed between hospitals with different market shares. Second, we added payment models interacted with time to the model to examine the influence of payment models. Payment models were added to the model using dummy variables, with FFS as the reference category. These analyses assessed whether the impact of A&F on LVC varied across hospitals with different payment models.

 Furthermore, the year 2020 was excluded from regression analyses, as hospital production fell significantly during the first year of the COVID-19 pandemic and all hospitals received a lumpsum payment during this period, thus no variation in payment models was observed. Analyses were performed for all seven LVC treatments combined and subsequently a subgroup analysis was performed for the low backpain treatment, as this was the only treatment which showed an increase in volume during the study period. All analyses were performed using the lme4 package in R [version 4.4].

## Results

###  Descriptives

 The proportion of LVC per hospital declined between 2017 and 2022, with the steepest decline during the COVID-19 pandemic (Figure 1). The total care volume also declined between 2017 and 2022, also with a decline during the COVID-19 pandemic (Figure 2). The proportion of LVC was lower in 2022 than in 2017 and the total care volume of 2022 was higher than in 2017.

**Figure 1:**
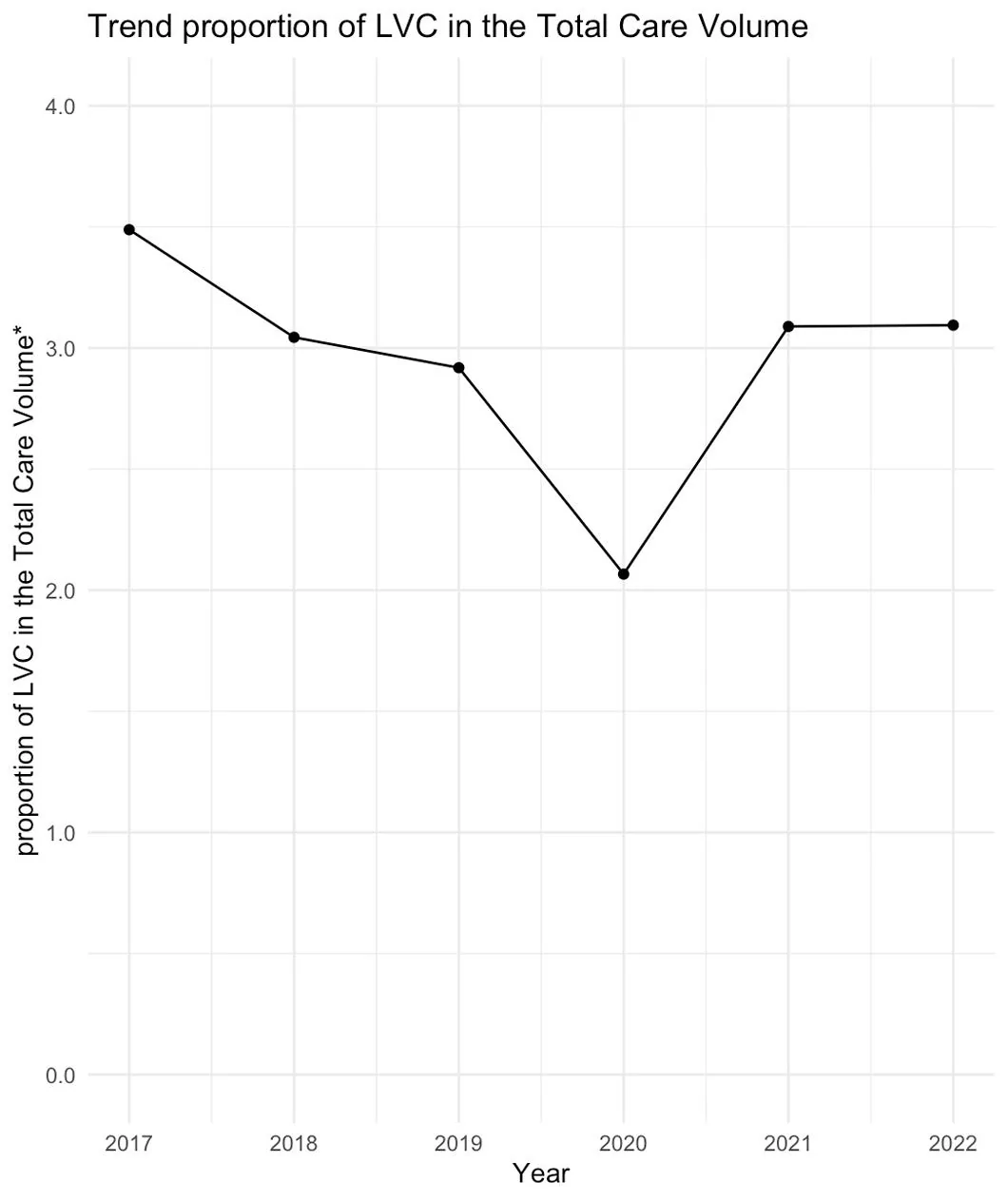
Trend Proportion Low-Value Care. Abbreviation: LVC, low-value care. * Proportion LVC/1000.

**Figure 2:**
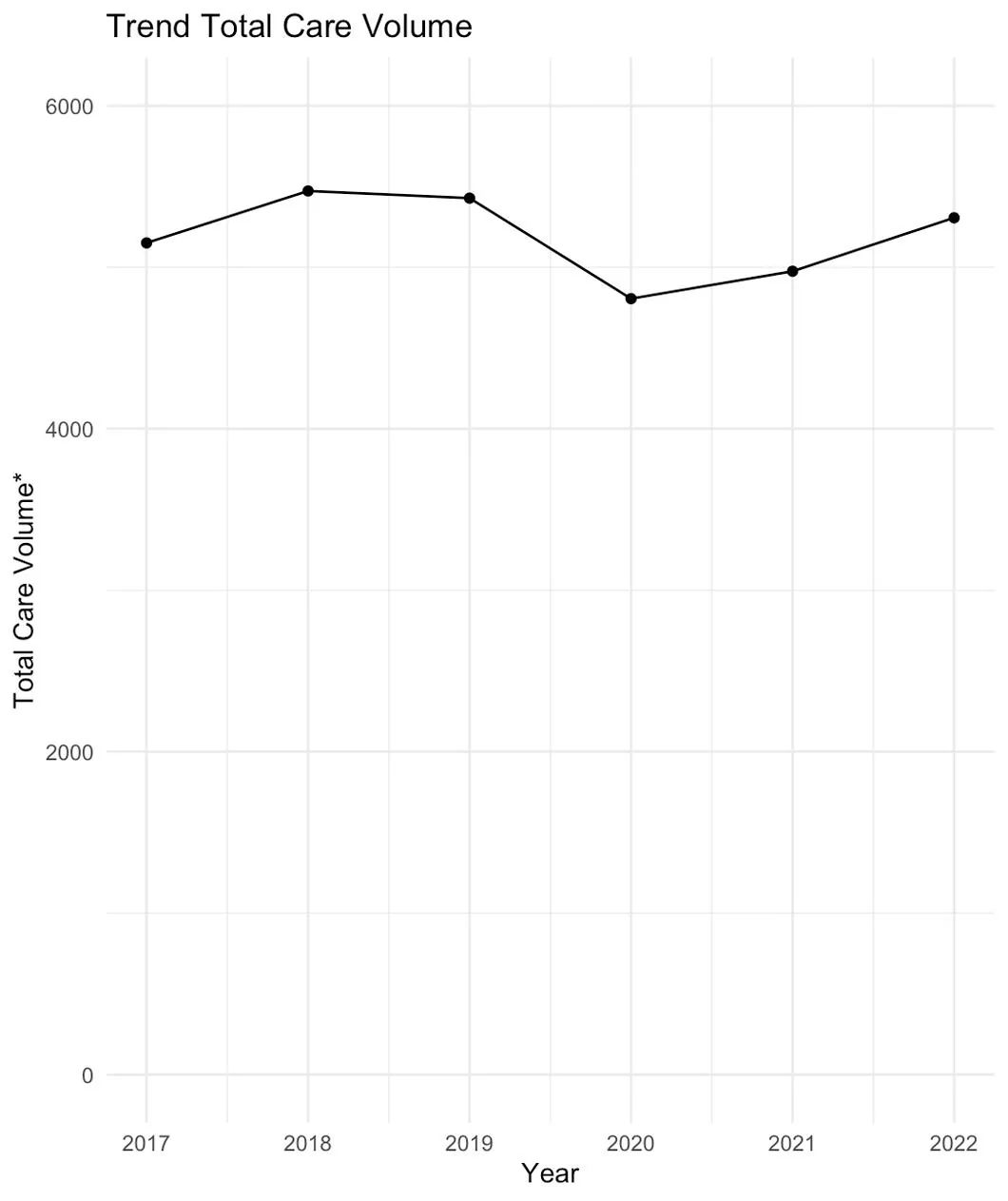
Trend Total Care Volume.

 In 2017,~18 thousandclaims of low-value were registered, over a total of 5.15 million claims. In 2022, ~16 thousand claims of LVC were registered over a total of 5.31 million claims. The proportion of LVC differed by a factor of 15 between the least and most LVC-providing hospitals (χ^2^ = 6.75, df = 1, *P* =.009).

 The decline in LVC was seen for six out of the seven treatments, only innervations and denervation techniques for low back pain showed an increase in the period 2017-2022 (Supplementary file 2).

 The type of payment model used in hospitals has shifted over the years. The GT model has become the most used in recent years (Table 2), while the CC and GB model were the most prevalent from 2017 to 2019. The FFS model was consistently the least used payment model. Over the 6-year study period no clear patterns in payment models were used for individual hospitals. Each hospital received a variety of payment models, with no single model being consistently applied.

**Table 2:** Descriptives

	**2017**	**2022**
Proportion LVC*, mean (SD)	5.33 (2.39)	4.68 (2.22)
Hospital care volume, mean (SD)	515.466 (425.270)	509.393 (393.393)
Payment model, No. (%)		
FFS	5 (7)	4 (6)
GT	12 (17)	47 (67)
CC	29 (42)	13 (19)
GB	23 (33)	6 (9)
Market share (Mean, SD)	0.302 (0.195)	0.282 (0.170)

Abbreviations: LVC, low-value care; SD, standard deviation; FFS, fee-for-service; GB, global budget; CC, cost ceiling; GT, gradual tariff. * A thousandth of a percentage point.

 The market share of the health insurer at hospitals was relatively constant over the years. In 60% of hospitals the health insurer had a market share between 0%-25%; in 30% of hospitals the health insurer had a market share of 26%-50% and in 10% the health insurer had a market share over 50%.

###  Trend in Low-Value Care 

 LVC showed a slight but statistically significant decline over the study period, with an OR of 0.98 (95% CI: 0.98–0.99). Hospitals with a higher insurer market share had greater odds of LVC at baseline (OR: 2.45; 95% CI: 1.57–3.80). Hospitals in which the insurers market share was higher exhibited a less pronounced decline in LVC over time, with an OR of 1.03 (95% CI: 1.00–1.05).

 In contrast to the other LVC treatments, the low back pain treatment showed an increase over time (OR: 1.13; 95% CI: 1.12–1.15). Market share was not significantly associated with baseline LVC use (OR: 1.47; 95% CI: 0.82–2.62). A significant interaction between time and market share (OR: 0.95; 95% CI: 0.92–0.98) indicated that the rate of increase in LVC over time was attenuated in hospitals with higher market share, suggesting that payer presence moderated the growth of LVC in this domain.

 The intraclass correlation coefficient (ICC) of 0.09 for the model of all treatments versus the ICC of 0.17 justifies the relevance of including the random intercept to account for hospital-level variation (Table 3).

**Table 3:** Trend in Low-Value Care

	**Proportion of All LVC Treatments**		**Proportion of Low Back Pain LVC Treatment **	
	**OR **	**95% CI **	**OR **	**95% CI **
Predictors				
Time trend^a^	0.98	0.98-0.99	1.13	1.12-1.15
Time trend × market share	1.03	1.00-1.05*	0.95	0.92-0.98*
Market share	2.45	1.57-3.80	1.47	0.82-2.62
Random effects				
s^2^	3.29		3.29	
t_00_	0.31		0.67	
ICC	0.09		0.17	
N_hospital_	70		69	
Observations	349		338	

Abbreviations: LVC, low-value care; OR, odds ratio; CI, confidence level; ICC, intraclass correlation coefficient. ^a^ Time after A&F intervention in years. * *P* < .05.

###  Trend in Low-Value Care Per Payment Model

 Only the CC model was associated with an increase in LVC (OR: 1.11; 95% CI: 1.08–1.14) (Table 4). The other payment models were associated with a decrease of LVC, of which the FFS model exhibited the smallest reduction in LVC (OR: 0.98; 95% CI: 0.98-0.99). The GB model was associated with the largest reduction in LVC (OR: 0.94; 95% CI: 0.91-0.97) and the GT model (OR: 1.01; 95% CI: 0.99-1.03) showed similar patterns in LVC to the FFS hospitals.

**Table 4:** Trend in Low-Value Care and Influence of Payment Model

	**Proportion of All LVC Treatments**		**Proportion of Low Back Pain LVC Treatment **	
	**OR **	**95% CI **	**OR **	**95% CI **
Predictors				
Time × Market Share^a^ (FFS default)	0.98	0.98-0.99*	1.13	1.12-1.14*
Time × market share × GB	0.94	0.91-0.97*	0.88	0.85-0.92*
Time × market share × GT	1.01	0.99-1.03	0.96	0.93-0.99*
Time × market share × CC	1.11	1.08-1.14*	1.05	1.02-1.09
GB	1.04	0.94-1.15	1.43	1.26-1.62*
GT	1.04	0.93-1.16	1.13	0.98-1.30
CC	0.84	0.75-0.94*	0.92	0.79-1.06
Market share	3.80	2.31-6.25*	2.33	1.24-4.39*
**Random effects**				
s^2^	3.29		3.29	
t_00_	0.33		0.67	
ICC	0.09		0.17	
N_hospital_	70		69	
Observations	349		338	

Abbreviations: LVC, low-value care; OR, odds ratio; CI, confidence level; ICC, intraclass correlation coefficient; FFS, fee-for-service; GB, global budget; CC, cost ceiling; GT, gradual tariff. ^a^ Time after A&F intervention in years, default is FFS payment model. * *P* < .05.

 For low back pain, the increase was highest in hospitals with a CC model (OR: 1.05; 95% CI: 1.02–1.09). Hospitals under the FFS model also showed a substantial yearly increase in LVC (OR: 1.13; 95% CI: 1.12–1.14), although the slope was less pronounced. By contrast, hospitals with GBs demonstrated a significantly attenuated trend (OR: 0.88; 95% CI: 0.85–0.92), and those with GT models also exhibited a weaker increase (OR: 0.96; 95% CI: 0.93–0.99).

## Discussion

 Payers in managed care settings are incentivized, and sometimes made responsible, to reduce LVC, potentially through interventions such as A&F at the provider level, with or without accompanying financial incentives.^24^ A large Dutch nationwide health insurer developed an A&F program specifically aimed at reducing LVC. For this the health insurer shared A&F on volume of 13 LVC treatments with the sales department of each Dutch hospital during the annual sales negotiations. Aim was to motivate hospitals to de-implement LVC in relative terms in their hospital. Analysis of the differences between hospitals suggests that the payer-initiated A&F intervention alone does not correlate with a lower proportion of LVC, using variation in hospital market share of our payer and its correlation to LVC. However, when A&F is paired with financial incentives to decrease production, there appears to be a meaningful association with reductions in LVC over time. Specifically, an FFS payment model, with a strong incentive for production showed the least reduction in LVC over time. Moreover, the weaker the incentive for production the stronger the effect of the A&F intervention over time. That is to say the GB model, with the weakest incentive for production, shows the largest decline in LVC.

 The GT model, which incentivizes production while imposing only a modest disincentive for exceeding the cap through reduced tariffs, exhibits a pattern similar to the FFS model. This similarity aligns with the theoretical foundations of both payment models, as both prioritize production incentives as a key mechanism. Furthermore, under a GT model the healthcare professional is not always aware whether a treatment decision falls under or over the cap, thus this model provides less direct financial incentives to change clinical behavior.

 The CC model showed an increase in LVC. While the CC model theoretically provides less incentive for LVC than FFS, this effect may be explained due to selection effects: hospitals known to provide high volumes of LVC may be more likely to receive a CC contract than an FFS model. These findings imply that the effectiveness of A&F may depend on the presence and structure of complementary financial mechanisms.

 Furthermore,lower back pain treatment represents a special category of LVC, distinguished by its high frequency and strong increase over time. At the beginning of the observed timespan, lower back pain accounted for 50% of all LVC cases (9199/18690), rising to over 80% (13506/16622) by the end. This upward trend in volume contrasts with the overall trajectory of other forms of LVC. Notably, differences in patterns between the application of A&F suggest a potentially stronger impact of A&F treatments on categories with increasing volumes of LVC. These findings indicate that A&F may be particularly effective in addressing areas where LVC is on the rise, although causality cannot yet be established. Further, the hospitals received a variety of payment models over the years, thus the effects found may present an underestimation of stable, multi-year contracts.

 In comparison with the existing literature, this study finds varying effects of A&F as a quality improvement intervention. While A&F is generally recognized as an effective strategy, its impact varies significantly across studies.^16^ The limited effect observed in this study may be partially attributed to suboptimal implementation within hospitals. Evidence suggests that A&F is most effective when it adheres to specific best practices: it should be delivered more than once; by a respected peer or supervisor; in both written and verbal formats; with explicit targets; and an accompanying action plan.^16^ Furthermore A&F is particularly impactful when baseline performance was low.^16^

 In this study, the health insurer delivered A&F annually to the sales departments of hospitals; however, it remains unclear whether and how this feedback was communicated to the relevant clinical departments. In addition, medical professionals did not always agree with either the objectives of de-implementation or the definitions of LVC. Subsequent discussions between the health insurer and medical professionals have in some cases led to adaptations of these definitions.

 Adapting the A&F intervention to align with these evidence-based criteria could enhance its effectiveness in driving the de-implementation of LVC. Additionally, the A&F provided by the health insurer was not coupled with direct financial incentives, such as specific bonuses for reducing a particular level of LVC. A&F combined with targeted financial incentives for the specific LVC in question may expedite the de-implementation process and enhance efforts to reduce LVC. Furthermore, the health insurer utilized claims-based A&F, which may be perceived as less reliable or actionable by healthcare professionals compared to patient record data or data derived from quality registries.^25,26^ This could influence the acceptance and perceived credibility of the feedback. Additionally, A&F may be less accepted when it is perceived as punitive.^27^ Providers may view A&F delivered by payers as a form of punitive oversight rather than a collaborative effort to improve care quality, potentially reducing their receptiveness to the intervention. Future research should explore healthcare providers’ attitudes toward payer-driven feedback to better understand these dynamics and identify strategies to enhance engagement, such as emphasizing collaborative goals and ensuring feedback is framed constructively.

 Our findings on payment models are consistent with existing literature examining the relationship between payment models and LVC. Previous studies have shown that payment models with weaker production incentives, such as the GB model, are associated with a lower likelihood of LVC compared to strong production-incentive models, such as FFS.^28-30^ Similarly, our study observed differing responses to GBs versus CC models, aligning with findings from Gaspar et al.^20^ GBs provide hospitals with a guaranteed budget, irrespective of their expenditure levels, effectively eliminating financial pressure to overproduce. In contrast, hospitals operating under CC models face strong incentives to produce slightly above the expenditure cap. This behavior is driven by the significant financial risk associated with failing to meet the cap, coupled with the relatively low marginal costs of exceeding it due to fixed salary costs. Slight overproduction provides hospitals with a CC financial security and enhances the hospital’s bargaining position for future contract negotiations.

 The CC and GT models are less described in literature, limiting opportunities for international comparison. However, regarding incentives the GT model closely resembles the FFS payment model, a similarity corroborated by our findings.

 While our study generally observed a decline in LVC that aligned with the payment incentives in use, the LVC treatment for non-specific back pain showed an increase during the study period. For this treatment, there may have been market changes such as a shift from independent treatment centres to hospitals. Furthermore, this suggests that the financial incentives may not be uniformly effective across all clinicians or departments. A possible explanation is that the payment models used in this study are high-level models applied broadly across all types of elective care and specialties within hospitals, without specific financial agreements targeting LVC treatments. As a result, not all healthcare providers may perceive or respond to the incentives to de-implement specific low-value treatments.

 Our study suggests that A&F, when aligned with appropriate hospital-level financial incentives, is significantly associated with a reduction in LVC over time. In the ongoing debate about the use of financial incentives to promote high-value care, complex models such as capitation and bundled payments are often proposed as alternatives to FFS reimbursement.^31,32^ While these models have shown promise in reducing LVC in certain contexts, they are frequently associated with complex, time-intensive negotiations, and the distribution of financial risk among stakeholders remains a key challenge.^29^ Additionally, methods like medical pre-authorization can create substantial barriers to care for patients. Our findings suggest that less complex hospital-level payment models such as the GB can effectively incentivize the reduction of LVC. Moreover, segmented payment models, where high-value care and LVC are reimbursed differently, may further accelerate the de-implementation of LVC.

 Moreover, other studies have highlighted that while LVC varies across payment models, the differences tend to be more pronounced for less expensive services, suggesting that financial incentives alone may not fully drive LVC provision.^33^ This underscores the need for multifaceted approaches that combine financial mechanisms with other strategies, such as clinician education or patient engagement, to effectively address the persistence of LVC. Integrating these complementary interventions may enhance the impact of payment models and ensure more comprehensive de-implementation efforts.^4^

###  Strengths and Limitations

 This study is, to our knowledge, the first to analyse the effect of a payer-initiated A&F program and advanced payment models on LVC. Strengths of the study include the use of a nationwide dataset from all hospitals in the Netherlands across multiple years, covering a broad range of LVC treatments.

 Several limitations should be noted. First, this was an observational study in which all hospitals received the A&F intervention without randomization of a control group, and data prior to the start of the intervention in 2016 were unavailable thus preintervention trends in LVC could not be assessed. Therefore, causal effects of A&F cannot be established. Second, data on hospital characteristics that may influence the selection of payment models and LVC provision (eg, growth strategies, financial position, regional role, staffing, or teaching status) were lacking.

 While these characteristics may confound analyses of payment models, it does not affect our estimate of the total trend in LVC. Third, this study utilized data from a single health insurer, and information on payment models used by other insurers was unavailable. As hospitals may be subject to multiple incentives depending on their payer mix, the observed impact of payment models is likely influenced more by the overall mix of payment arrangements than by any single model. Fourth, the criteria for what was considered LVC changed for 5/7 treatments during the study period. Although such changes may introduce misclassification, the low incidence of affected indications and consistent results from a sensitivity analysis of light therapy suggest minimal influence on our overall findings.

 Furthermore, the analysis focused on a specific set of LVC treatments. While this was a diverse set of treatments, trends in LVC and the impact of payment models may differ between treatments, such as between high-cost versus low-cost treatments. Moreover, data on high-value care per diagnosis were not available, which prevented subgroup analyses of the impact of payment models on different treatment types. Lastly, this study was limited to seven LVC treatments; as numerous additional LVC treatments are likely to be present in hospital settings, the estimated proportion of LVC represents an underestimation though the relative patterns observed remain informative for understanding broader trends in LVC de-implementation.

## Conclusion

 A payer-initiated A&F intervention alone was not associated with a reduction of LVC in hospitals. However, when A&F was combined with a payment model that stimulates de-implementation, a significant reduction in LVC over time was observed. The GB Model provides hospitals with the lowest financial incentives for production, making it a promising approach for reducing LVC. To effectively reduce LVC, it may help to align financial incentives with de-implementation objectives.

## Disclosure of artificial intelligence (AI) use

 Not applicable.

## Ethical issues

 Ethical approval and consent to participate Study methods were carried out following the Declaration of Helsinki and other relevant regulations and guidelines. Conditions are fulfilled under which the use of electronic health records for research purposes in the Netherlands is allowed. Under these conditions, neither informed consent from study subjects nor approval by a medical ethics committee is obligatory for this type of observational study, containing no directly identifiable data (art. 24 GDPR Implementation Act jo art. 9.2 sub j GDPR). Only de-identified data on the level of providers was obtained, to protect the privacy of hospitals practices, an agreement on the handling of data was signed.

## Conflicts of interest

 Authors declare that they have no conflicts of interest.

## Data availability statement

 The data that support the findings of this study are available from the health insurer which were used under license for the current study and so are not publicly available. Data at the aggregated level are available from the corresponding author upon reasonable request and with the permission of the health insurer.

## Endnotes


^[1]^ LVC is identified by the health insurer as a type of treatment that is low-value for a certain patient population, with a specific diagnosis. Data obtained in this study did not include “high-value care” ie, the high value treatments performed for patients with the same diagnosis. Therefore, we used the volume of LVC as the numerator and the total care volume as the denominator in this study. The total care volume can include high-value care and other types of LVC, ie, other treatments than the 7 LVC treatments in this study.

## Supplementary files


Supplementary file 1. List of Excluded Low-Value Care Treatments.

Supplementary file 2. Low-Value Care Trend Per Treatment Type.
