# Supplementary material for: Trends in Low-Value Care Following a Payer-Initiated Audit and Feedback Intervention: A Retrospective Analysis in Hospitals With Varying Payment Models
Source: Int J Health Policy Manag. 2026 Aug 18;15:9060. doi: 10.34172/ijhpm.9060 (PMC13770233; doi:10.34172/ijhpm.9060)
Supplement: Supplementary file 1 [file ijhpm-15-9060-s001.pdf]

Article title: Trends in Low-Value Care Following a Payer-Initiated Audit and Feedback Intervention: A Retrospective Analysis in Hospitals with Varying Payment Models

Journal name: International Journal of Health Policy and Management (IJHPM)

Authors' information: Vera de Weerdt[1,2]*, Xander Koolman[1], Hanna C. Willems[3,4], Sjoerd Repping[2,5], Piet A. Stam[1], Jacques van Limbeek[6], Eric J.E. van der Hijden[1,7]

[1]Department of Health Economics, School of Business and Economics, Vrije Universiteit, Amsterdam, The Netherlands.

[2]Amsterdam University Medical Center, Amsterdam, The Netherlands.

[3]Department of Geriatrics, Amsterdam University Medical Center, Amsterdam, The Netherlands.

[4]Leading the Change, Zorgevaluatie Nederland, The Netherlands.

[5]National Healthcare Institute, Diemen, The Netherlands.

[6]ONVZ Health Insurance, Houten, The Netherlands.

[7]Zilveren Kruis Health Insurance, Zeist, The Netherlands.

***Correspondence to:** Vera de Weerdt; Email: v.de.weerdt@vu.nl

Citation: de Weerdt V, Koolman X, Willems HC, et al. Trends in low-value care following a payer-initiated audit and feedback intervention: a retrospective analysis in hospitals with varying payment models. Int J Health Policy Manag. 2026;15:9060. doi:10.34172/ijhpm.9060

Supplementary file 1. List of Excluded Low-Value Care Treatments

Table Sq. Description of LVC treatments classified by the health-insurer, which were excluded from analyses

	LVC treatment	For indication (diagnosis):
1	Spinal fusion	Chronic a-specific low back pain, unless:<br>- prior maximal conservative therapy<br>- spondylolisthesis symptomatic > grade 1, increasing with dynamic X-ray
2	Specialist rehabilitation	Chronic pain and psychiatric disorders unless:<br>- pain classified grade 3 according to Dutch Workgroup Pain rehabilitation (WPN) or<br>- in rare cases: pain classified grade 4 according to Dutch Workgroup Pain rehabilitation (WPN) Where it is clearly justified that there is multiple, complex, and highly complex pathology, involving interaction between treatment goals and/or severe functional impairments, and where all other, less costly treatment options have been exhausted without sufficient effect.
3	Hyperbaric Oxygen Therapy	Other diagnosis than approved by the Dutch Healthcare Institute
4	Cardiac rehabilitation	Stable Angina
5	Surgical treatment	Stable Angina
6	Pulmonary rehabilitation	Chronic obstructive pulmonary disease

*These treatments were performed in <50% of hospitals, thus were excluded from statistical analyses.
