# Supplementary material for: Trends in Low-Value Care Following a Payer-Initiated Audit and Feedback Intervention: A Retrospective Analysis in Hospitals With Varying Payment Models
Source: Int J Health Policy Manag. 2026 Aug 18;15:9060. doi: 10.34172/ijhpm.9060 (PMC13770233; doi:10.34172/ijhpm.9060)
Supplement: Supplementary file 2 [file ijhpm-15-9060-s002.pdf]

Article title: Trends in Low-Value Care Following a Payer-Initiated Audit and Feedback Intervention: A Retrospective Analysis in Hospitals with Varying Payment Models

Journal name: International Journal of Health Policy and Management (IJHPM)

Authors' information: Vera de Weerdt[1,2]*, Xander Koolman[1], Hanna C. Willems[3,4], Sjoerd Repping[2,5], Piet A. Stam[1], Jacques van Limbeek[6], Eric J.E. van der Hijden[1,7]

[1]Department of Health Economics, School of Business and Economics, Vrije Universiteit, Amsterdam, The Netherlands.

[2]Amsterdam University Medical Center, Amsterdam, The Netherlands.

[3]Department of Geriatrics, Amsterdam University Medical Center, Amsterdam, The Netherlands.

[4]Leading the Change, Zorgevaluatie Nederland, The Netherlands.

[5]National Healthcare Institute, Diemen, The Netherlands.

[6]ONVZ Health Insurance, Houten, The Netherlands.

[7]Zilveren Kruis Health Insurance, Zeist, The Netherlands.

***Correspondence to:** Vera de Weerdt; Email: v.de.weerdt@vu.nl

Citation: de Weerdt V, Koolman X, Willems HC, et al. Trends in low-value care following a payer-initiated audit and feedback intervention: a retrospective analysis in hospitals with varying payment models. Int J Health Policy Manag. 2026;15:9060. doi:10.34172/ijhpm.9060

Supplementary file 2. Low-Value Care Trend Per Treatment Type

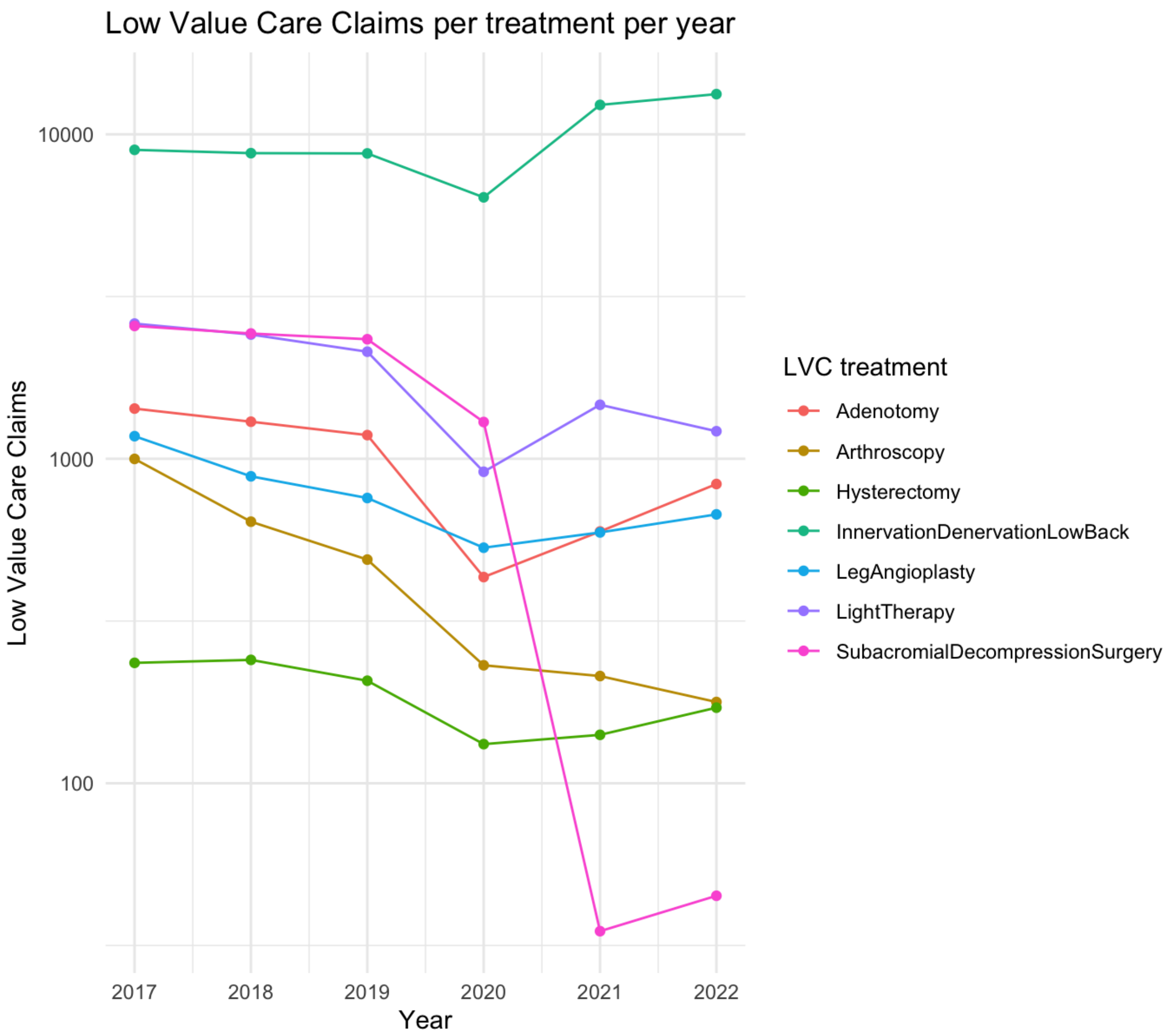

Figure S1. Volume LVC treatments per type from 2017-2022
